# What the Public Forget

**Published:** 1920-05-01

**Authors:** 


### THE NEW WORLD AND THE VOLUNTARY HOSPITAL.

### What the Public Forget.

During the war the prophets promised us a new heaven and a new earth.

I think I am correct when I say that the new earth has arrived. I am quite willing to allow the new heaven to stand over for the present. The old one indeed may not be so bad as to demand supersession. I know a good many people who look back with a sigh of regret to the old earth with all its earthiness. It may be an ill-considered judgment to be revised with riper experience. But the facts are as stated. The war has wrought a social revolution in the heart of Merrie England, and it has come about so silently that people are only beginning to realise what has taken place. The rich have become poor, and the poor have become rich! I cannot prophesy anything about the new heaven, but this is one aspect of the new earth.

There is probably no British institution which this affects, and will affect, more intimately and more seriously than the voluntary hospital. At the present moment hundreds of voluntary hospitals are in a state of almost hopeless debt. The fact is deplorable. But, likewise, it is absolutely and perfectly natural. I have hammered Hospital Committees full many a time and oft for their attitude of detachment and aloofness where the conditions of employment of nurses were concerned. I do not go so far as to say that I have no words left, for I shall probably return to the attack. On the other hand, I think that with regard to the destitute poor Hospital Committees have displayed a spirit of patriotism second to none.

In the blackest days of the war—and we had some black days—the voluntary hospitals "carried on." The sick poor thronged their gates, and they always found them open. Physician, surgeon, and nurse stood at their posts, while the governors undertook the responsibility of the financial problem. Do you remember it, friend reader? It is hard to get back, even for a moment, to war conditions, to the strain and the tension and the terror. Our one only and supreme duty was to "carry on."

The hospitals carried on. To have done otherwise would have been, commercially, sound business, but, at the same time, playing the enemy game. What a headline for enemy propaganda—"English Hospitals Closed." The English hospitals never at any time thought of closing. They realised all the time that they were obeying the Empire clarion-call. They drew a Bill on the nation which they expected to be honoured on maturity.

All this happened on the old earth. Now we have a new earth. The war is over. We have achieved Victory, and the black days are dead. But, in the meantime, things have changed. The rich have become poor and the poor have become rich.

I happened to be travelling to town a morning or two ago, and opposite to me in the train were two business men. They were reading their morning papers, and one of them drew the attention of his friend to a hospital appeal. He waxed quite warm on the bad business methods and general asleepness of hospitals, his point being that the working classes are now thoroughly well able to pay. A new earth argument, which is perfectly sound.

This man is a type of the general public. What he either forgot or failed to realise is that during the war the new earth had not come, and that in those days a debt of honour was contracted by the nation. This needs driving home. There is, I think, a common consensus of opinion that for the future new rules and regulations will have to be formulated, and that the working classes will have to maintain the hospitals. I have no doubt that they will be quite ready to do so when they are properly informed. They cannot, however, be expected to liquidate the debts that have already accrued. Personally I regard the change that has taken place as an item in the progress of civilisation. For one section of the population to look to another section for a constant flow of charity is demoralising. Poverty in the future will doubtless exist, but, let it be hoped, as an exception to the general rule. In certain circumstances poverty is a crime, and should be so regarded and punished. It will add to the working man's dignity to know that he is paying for his treatment in sickness.

The point of my remarks is that the public require instruction. It must be demonstrated to them that their first duty is to redeem the war debt, and this information should be accompanied by a promise that in the future similar appeals are likely to be very much less frequent, if not altogether to cease. At the same time a campaign of propaganda should be carried into the industrial world. The benevolent employer will almost certainly withdraw or greatly reduce his subscriptions. The labourer cannot expect to have it every way. His demand for high wages has been peremptory, and has been granted. An appeal for benevolence is not following suit. The labourer must learn the lesson of self-reliance. At the same time the cost of carrying on when the country was in the grip of war must be liquidated. IERNE.

